# α2-Adrenergic Receptors in Hypothalamic Dopaminergic Neurons: Impact on Food Intake and Energy Expenditure

**DOI:** 10.3390/ijms26083590

**Published:** 2025-04-10

**Authors:** Byong Seo Park, Hye Rim Yang, Hara Kang, Kwang Kon Kim, Yang Tae Kim, Sunggu Yang, Jae Geun Kim

**Affiliations:** 1 Division of Life Sciences, College of Life Sciences and Bioengineering, Incheon National University, Incheon 22012, Republic of Korea; 2021s135@inu.ac.kr (B.S.P.); hr.yang0414@gmail.com (H.R.Y.); harakang@inu.ac.kr (H.K.); rabit7890@gmail.com (Y.T.K.); 2 Research Center of Brain-Machine Interface, Incheon National University, Incheon 22012, Republic of Korea; 3 Division of Gastroenterology and Hepatology, Department of Medicine, Stanford University, Stanford, CA 94305, USA; kwangkon@stanford.edu; 4 Department of Nano-Bioengineering, Incheon National University, Incheon 22012, Republic of Korea

**Keywords:** alpha-2 adrenergic receptor, hypothalamic dopamine neurons, food intake, energy expenditure

## Abstract

The adrenergic system plays an active role in modulating synaptic transmission in hypothalamic neurocircuitry. While α2-adrenergic receptors are widely distributed in various organs and are involved in various physiological functions, their specific role in the regulation of energy metabolism in the brain remains incompletely understood. Herein, we investigated the functions of α2-adrenergic receptors in the hypothalamus on energy metabolism in mice. Our study confirmed the expression of α2-adrenergic receptors in hypothalamic dopaminergic neurons and assessed metabolic phenotypes, including food intake and energy expenditure, after treatment with guanabenz, an α2-adrenergic receptor agonist. Guanabenz treatment significantly increased food intake (0.25 ± 0.03 g vs. 0.98 ± 0.05 g, *p* < 0.001) and body weight (−0.1 ± 0.04 g vs. 0.33 ± 0.03 g, *p* < 0.001) within 6 h post-treatment. Furthermore, guanabenz markedly elevated energy expenditure parameters, including respiratory exchange ratio (RER, 1.017 ± 0.007 vs. 1.113 ± 0.03, *p* < 0.01) and carbon dioxide production (1.512 ± 0.018 mL/min vs. 1.635 ± 0.036 mL/min, *p* < 0.05), compared to vehicle-treated controls. Furthermore, using chemogenetic techniques, we demonstrated that the altered metabolic phenotypes induced by guanabenz treatment were effectively reversed by inhibiting the activity of dopaminergic neurons in the hypothalamic arcuate nucleus (ARC) using a chemogenetic technique. Our findings suggest functional connectivity between hypothalamic α2-adrenergic receptor signals and dopaminergic neurons in metabolic controls.

## 1. Introduction

The adrenergic system plays a pivotal role as a modulator of synaptic transmission in both the central and peripheral nervous systems. Endogenous catecholamines, such as epinephrine and norepinephrine, activate various G protein-coupled receptors (GPCRs) within neurons, including α1, α2, and β-adrenergic receptors. Among these, α2-adrenergic receptors are composed of approximately 450 amino acids and are associated with Gi proteins, thus reducing adenylyl cyclase activity and promoting the efflux of extracellular potassium, leading to membrane hyperpolarization [1,2]. These receptors are widely distributed in various organs and contribute to various physiological processes, including regulation of neurotransmitter release, vascular blood pressure control, insulin secretion, and fat metabolism [3,4].

Research on the association between adrenergic receptors and appetite regulation has focused primarily on the dorsal raphe (DR) nucleus, which is involved in numerous physiological functions, such as the sleep-wake cycle, motivation, pain modulation, energy balance, and feeding behaviors [5,6,7]. Studies have shown that while the administration of phenylephrine, an α1-adrenergic receptor agonist, into the DR of rats does not significantly affect appetite, clonidine, an α2-adrenergic receptor agonist, notably enhances appetite [8,9]. Specifically, certain areas within the hypothalamus, including the ventromedial area, exhibit a sensitive response to α-agonists, resulting in increased appetite. Furthermore, in other hypothalamic areas, such as the paraventricular nucleus, α2-adrenergic receptor agonist stimulates appetite, while activation of α1-adrenergic receptors suppresses it [8,9]. These findings suggest different functions for α2-adrenergic receptors and α1-adrenergic receptors in the hypothalamic regulation of appetite.

Concurrently, dopamine, the primary catecholamine in the brain, plays a crucial role in a variety of neural functions, including regulation of the neuroendocrine system, locomotion, cognitive processes, emotional behavior, memory, and reward perception [10,11,12,13]. In particular, dopamine serves as an active neurotransmitter that modulates feeding motivation and energy expenditure. Recent evidence has highlighted the dynamic involvement of hypothalamic dopaminergic neurons in the regulation of circuit activity within the hypothalamus, projecting to key regions, such as the arcuate nucleus (ARC) and median eminence, thus influencing neurons responsible for feeding behaviors [14,15].

Pharmacological investigations using D1- and D2-like receptor agonists and antagonists have provided information on the modulation of dopamine receptor activity in the hypothalamus, which effectively regulates food intake [16,17]. However, the precise mechanisms underlying the activation of hypothalamic dopaminergic neurons and their role in the regulation of food intake remain unclear. Moreover, discrepancies in the findings of various studies that examine the impact of dopaminergic signaling on food intake may arise from the various effects of the dopaminergic pathway on different hypothalamic regions or the involvement of different receptor subtypes.

The present study aims to investigate the role of α2-adrenergic receptors in the regulation of feeding behavior and energy expenditure through their interaction with hypothalamic dopaminergic neurons. Specifically, we explore how the activation of α2-adrenergic receptors by guanabenz, an α2-adrenergic receptor agonist, influences food intake, body weight, and energy expenditure in mice. Furthermore, we examine the impact of inhibiting dopaminergic neuronal activity on these metabolic effects to elucidate the functional relationship between α2-adrenergic signaling and hypothalamic dopaminergic neurons. To achieve these objectives, we employed a combination of pharmacological treatments, chemogenetic techniques for neuronal inhibition, electrophysiological analyses to assess neuronal activity, and metabolic measurements, including food intake, body weight monitoring, and indirect calorimetry to evaluate energy expenditure.

## 2. Results

### 2.1. Adrenergic Signals Modulate Feeding Behavior by Targeting Hypothalamic Dopaminergic Neurons

**Figure 1:**
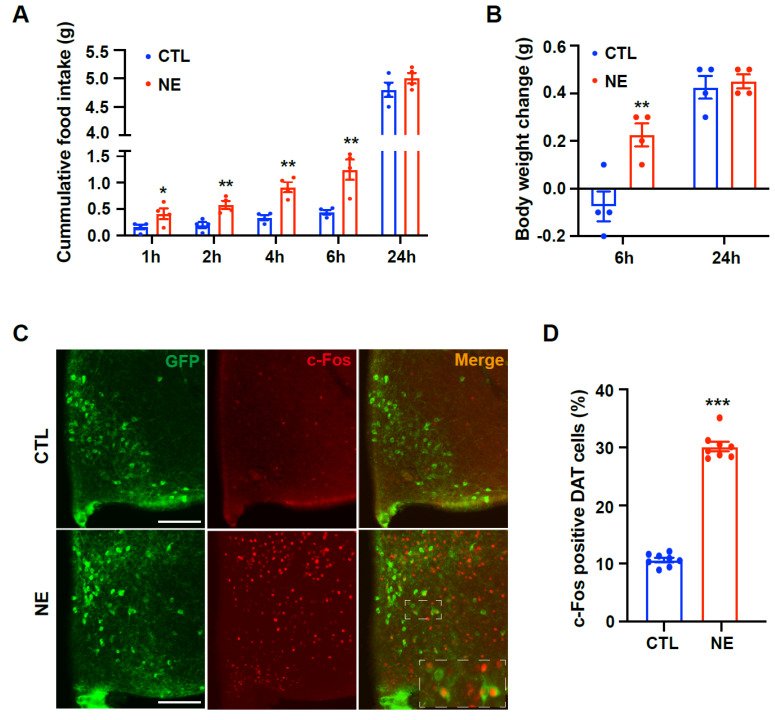
Hypothalamic dopaminergic neurons are activated by norepinephrine. Food intake is measured after intracerebroventricular (i.c.v.) injection of norepinephrine into C57BL/6 mice. (**A**) The cumulative food intake increases significantly in norepinephrine-treated mice compared to control mice for 6 h after injection (n = 4/group). (**B**) Norepinephrine treatment increased body weight compared to control mice during the 6-h period (n = 4/group). (**C**,**D**) Norepinephrine-induced activation of dopaminergic neurons in ARC is determined by assessing changes in c-Fos immunoreactivity in the hypothalamus of DAT-Cre; GFP mice. (**C**) Representative photographs (higher magnification image in box) and (**D**) graphs showing enhanced c-Fos activity in DAT-positive neurons induced by norepinephrine treatment (n = 8 sections/4 mice/group). Results are presented as mean ± SEMs. (**A**,**B**,**D**): * *p* < 0.05, ** *p* < 0.01, *** *p* < 0.001 vs. CTL, as determined using an unpaired two-tailed Student’s *t*-test. CTL (Control); NE (Norepinephrine). Scale bar = 100 μm.

To confirm the impact of altered adrenergic signaling on feeding behavior, we assessed food intake after intracerebroventricular (i.c.v.) administration of norepinephrine in C57BL/6 mice. After injection, norepinephrine treatment resulted in increased food intake, showing approximately a three-fold rise (0.45 ± 0.04 g vs. 1.25 ± 0.19 g, *p* < 0.01) compared to vehicle-treated mice for 6 h (Figure 1A). However, the two groups had no discernible difference in 24-h food intake (4.80 ± 0.13 g vs. 5.00 ± 0.09 g) (Figure 1A). Mice treated with norepinephrine exhibited an increase in body weight (−0.08 ± 0.06 g vs. 0.23 ± 0.07 g, *p* < 0.01) during the initial 6-h period, but no significant alteration was observed in 24-h body weight change (0.43 ± 0.05 g vs. 0.45 ± 0.03 g) (Figure 1B). Furthermore, we evaluated the activity of dopaminergic neurons in response to norepinephrine treatment by quantifying c-Fos immunosignals in hypothalamic dopaminergic neurons (Figure 1C). In particular, c-Fos-positive dopaminergic neurons were elevated in the hypothalamic arcuate nucleus (ARC) of norepinephrine-treated mice (Figure 1D). These findings suggest a potential connection between adrenergic signals in hypothalamic dopaminergic neurons and the regulation of energy metabolism in the hypothalamus.

### 2.2. α2-Adrenergic Receptors Regulate the Activity of Dopaminergic Neurons in the Hypothalamus

**Figure 2:**
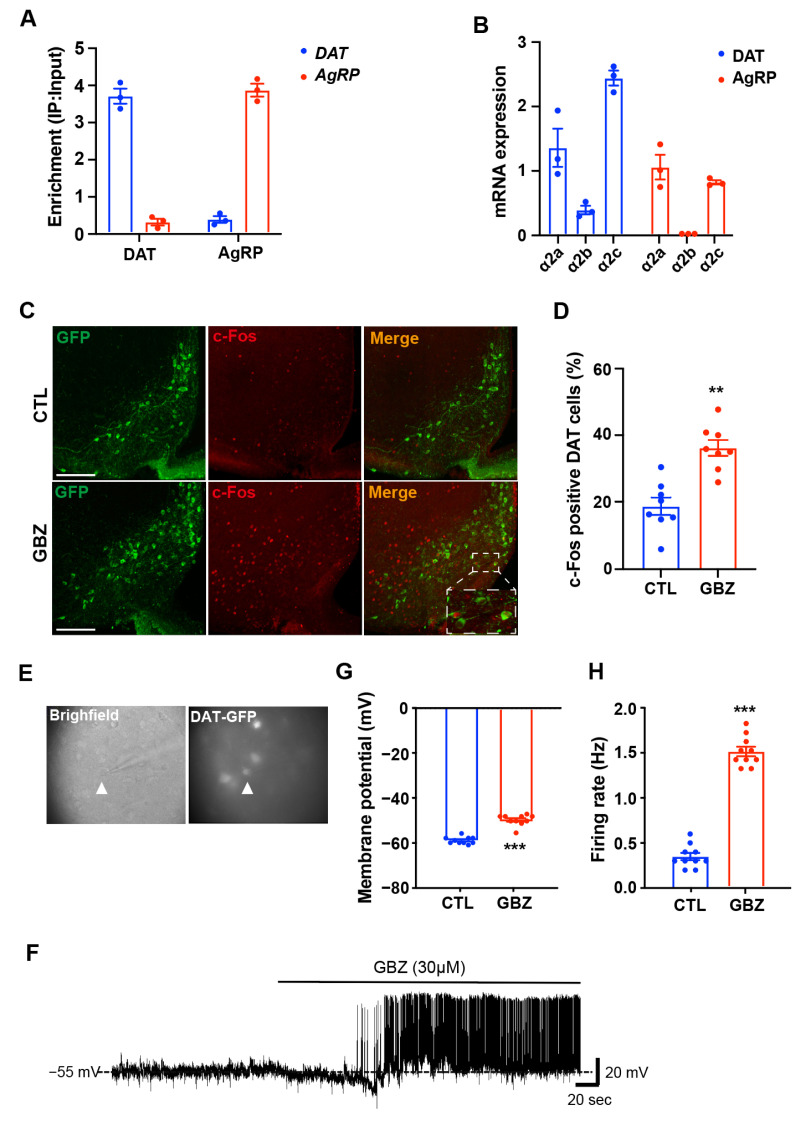
Treatment of α2-adrenergic receptor agonists enhances the activity of dopaminergic neurons in the hypothalamic ARC. (**A**) qPCR data confirmed the enrichment of DAT or AgRP mRNA expression in RNA purified from hypothalamic DAT- or AgRP-positive neurons using Ribo-tag technique (n = 3/group). (**B**) α2A, α2B, and α2C subtypes of the α2-adrenergic receptor were expressed in DAT-positive or AgRP neurons (n = 3/group). (**C**) Representative images show c-Fos immunosignals, a molecular marker of neuronal activity, in the hypothalamic arcuate nucleus (ARC) of mice treated with vehicle or guanabenz, as determined by immunohistochemistry (higher magnification image in box). (**D**) The number of c-Fos-positive cells increased significantly in DAT-positive neurons within the hypothalamic ARC of guanabenz-treated mice (n = 8 sections/4 mice/group). (**E**) The bright-field and fluorescent illumination of DAT-GFP neurons are attached to a whole-cell recording pipette (the arrowhead indicates the target cell). (**F**) Treatment with guanabenz produced high-frequency spikes in DAT-positive neurons. (**G**) The resting membrane potential and the (**H**) firing rate increase with guanabenz treatment (n = 10 cells/5 mice/group). Results are presented as means ± SEMs. (**D**,**G**,**H**): ** *p* < 0.01, *** *p* < 0.001 vs. CTL, as determined using an unpaired two-tailed Student’s *t*-test. CTL (Control); GBZ (Guanabenz). Scale bar = 100 μm.

Norepinephrine acts as an endogenous ligand for various adrenergic receptors in the brain, including the α1 and α2 subtypes, as well as the β1 and β2 subtypes. Given our focus on elucidating the role of α2-adrenergic receptors in the hypothalamic control of energy metabolism, we verified the expression of α2-adrenergic receptors in hypothalamic dopaminergic neurons using the Ribo-Tag technique with DAT-Cre; Rpl22^HA^ mice or AgRP-Cre; Rpl22^HA^ mice, which express the HA-tagged ribosomal protein Rpl22 in DAT- or AgRP-positive neurons. We validated the purification of mRNA extracted from hypothalamic dopaminergic neurons by confirming the enrichment of DAT and AgRP mRNA in the purified sample compared to the input control sample (Figure 2A). Furthermore, we confirmed the expression of α2A, α2B, and α2C subtypes of the α2-adrenergic receptor in purified mRNA from DAT-positive neurons (Figure 2B). To assess the active role of α2-adrenergic receptors in hypothalamic dopaminergic neurons, we evaluated changes in dopaminergic neuron activity by i.c.v. injection of the α2 adrenergic receptor agonist, guanabenz. Significantly elevated levels of c-Fos, a molecular marker of neuronal activity, by guanabenz treatment, were observed in the ARC of guanabenz-treated mice compared to control mice (Figure 2C,D). Furthermore, we observed the activation of dopaminergic neurons in the ARC of guanabenz-treated mice compared to control mice through whole-cell patch-clamp recordings, with DAT-positive neurons retaining GFP signals (Figure 2E). Dopaminergic neurons were strongly depolarized in response to guanabenz treatment (Figure 2F), with an elevation in the resting membrane potential and the firing rate by guanabenz (Figure 2G,H). These results suggest that dopaminergic neurons are governed by α2-adrenergic receptors within the hypothalamus.

### 2.3. Central Administration of Guanabenz Leads to Increased Food Intake and Energy Expenditure

**Figure 3:**
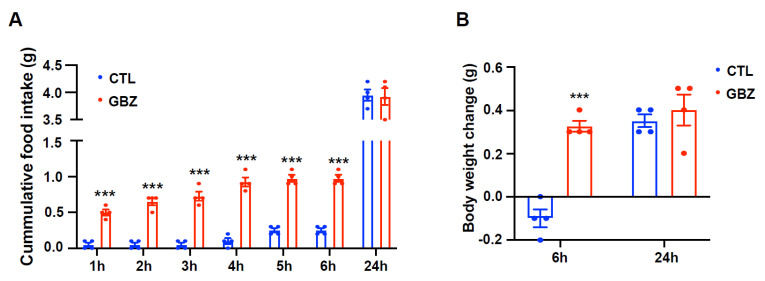
Guanabenz-treated mice show increased food intake and body weight. Food intake and body weight are measured after intracerebroventricular (i.c.v.) injection of guanabenz into C57BL/6 mice (n = 4/group). (**A**) The cumulative food intake of guanabenz-treated mice is significantly higher than that of vehicle-treated control mice 6 h post-injection, with no alteration observed in 24-h food intake. (**B**) Guanabenz treatment led to an increase in body weight compared to vehicle-treated control mice at 6 h post-injection, with no significant alteration observed in 24 h changes in body weight. Results are presented as mean ± SEMs. (**A**,**B**): *** *p* < 0.001 vs. CTL, as determined using an unpaired two-tailed Student’s *t*-test. CTL (Control); GBZ (Guanabenz).

**Figure 4:**
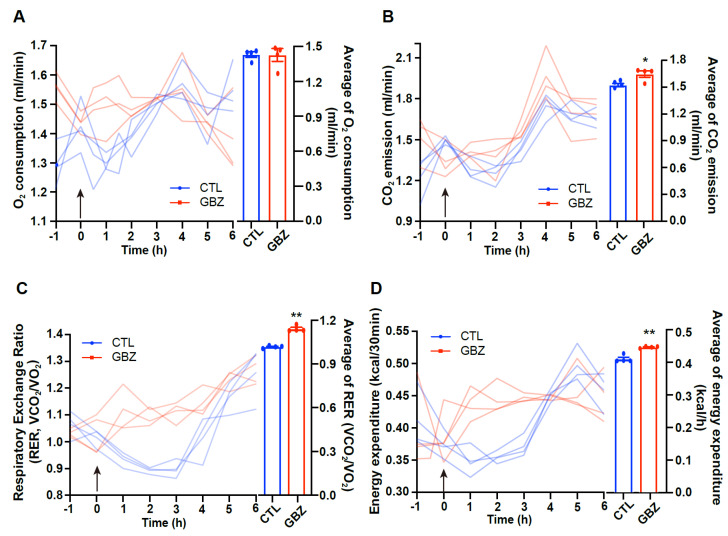
Guanabenz-treated mice exhibit an increase in energy expenditure. Metabolic phenotypes were evaluated after guanabenz injection in C57BL/6 mice using indirect calorimetry (n = 4/group). (**A**) Oxygen consumption (VO_2_) did not show significant differences, but (**B**) carbon dioxide production (VCO_2_), (**C**) respiratory exchange ratio (RER), and (**D**) energy expenditure increased in mice treated with guanabenz compared to vehicle-treated control mice over a 6-h period. Results are presented as mean ± SEMs. A-D: * *p* < 0.05, ** *p* < 0.01 vs. CTL, as determined using an unpaired two-tailed Student’s *t*-test. The arrow indicates the time of the guanabenz injection. CTL (Control); GBZ (Guanabenz).

To evaluate the impact of α2 adrenergic receptor activation on metabolic phenotypes, we assessed food intake and body weight after i.c.v. administration of guanabenz in C57BL/6 mice. We observed a significant increase in cumulative food intake (0.25 ± 0.03 g vs. 0.98 ± 0.05 g, approximately four-fold increase, *p* < 0.001) in guanabenz-treated mice compared to vehicle-treated control mice over a 6-h period post-injection, although there was no disparity in 24-h food intake (4.10 ± 0.17 g vs. 4.04 ± 0.17 g) (Figure 3A). Furthermore, mice treated with guanabenz showed body weight gain (−0.1 ± 0.04 g vs. 0.33 ± 0.03 g, *p* < 0.001) over a 6-h duration, but there was no difference in 24-h body weight change (0.34 ± 0.05 g vs. 0.40 ± 0.05 g) (Figure 3B). To investigate whether α2 adrenergic receptor activation influenced energy expenditure, we analyzed metabolic parameters, including oxygen consumption (VO_2_), carbon dioxide production (VCO_2_), respiratory exchange ratio (RER), and energy expenditure, using an indirect calorimetry system. Although there were no significant differences in VO_2_ (1.429 ± 0.025 mL/min vs. 1.427 ± 0.055 mL/min) during the 6-h post-guanabenz treatment period, VCO_2_ was elevated in guanabenz-treated mice compared to vehicle-treated controls (1.512 ± 0.018 mL/min vs. 1.635 ± 0.036 mL/min, *p* < 0.05) (Figure 4A,B). Furthermore, RER, which reflects the ratio of oxygen consumption to carbon dioxide production (1.017 ± 0.007 vs. 1.113 ± 0.028, *p* < 0.01) and energy expenditure (0.408 ± 0.006 kcal/h vs. 0.444 ± 0.001 kcal/h, *p* < 0.01), increased markedly in guanabenz-treated mice during the 6 h after injection (Figure 4C,D). These findings indicate the involvement of α2-adrenergic receptors in the regulation of energy metabolism, including food intake and energy expenditure.

### 2.4. Reducing Dopaminergic Neuron Activity Mitigated the Increase in Food Intake and Energy Expenditure Induced by Guanabenz

**Figure 5:**
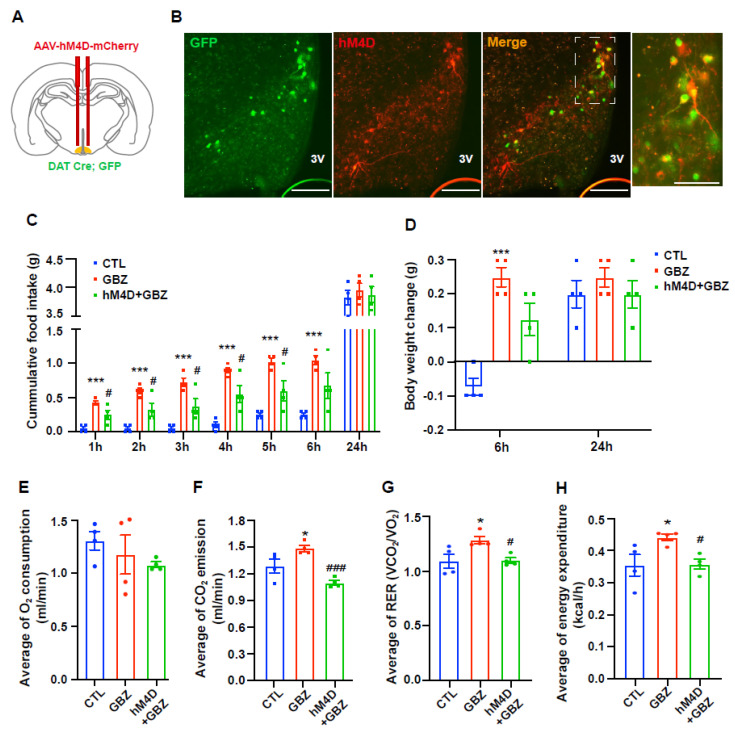
Guanabenz-induced increase in food intake and energy expenditure was rescued by the inactivation of dopaminergic neurons in ARC. (**A**) The AAV-hSyn-DIO-hM4D (Gi)-mCherry (hM4D) virus is injected into the hypothalamic arcuate nucleus (ARC) of DAT-cre; GFP mice to inhibit the activity of dopaminergic neurons. (**B**) Representative images show the hM4D virus (green) in DAT-positive neurons (red) in hypothalamic ARC. The insets show the signals at higher magnifications. (**C**) The increased food intake induced by guanabenz is decreased in hM4D virus-injected mice (n = 4/group). (**D**) No significant changes in body weight occurred in guanabenz-treated mice after the inactivation of dopaminergic neurons in the hypothalamic ARC (n = 4/group). (**E**) In all groups, there were no significant differences in oxygen consumption (VO_2_). The guanabenz-induced elevation in (**F**) carbon dioxide production (VCO_2_), (**G**) respiratory exchange ratio (RER), and (**H**) energy expenditure was almost completely rescued by injection of the hM4D virus during the 6-h post-guanabenz treatment period. Results are presented as mean ± SEMs. C-H: * *p* < 0.05, *** *p* < 0.001 vs. CTL; # *p* < 0.05, ### *p* < 0.001 vs. GBZ, as determined using a one-way ANOVA followed by Sidak’s post hoc multiple comparison test. CTL (Control); GBZ (Guanabenz); hM4D (AAV-hSyn-DIO-hM4D). Scale bar = 100 μm.

To investigate the involvement of hypothalamic dopaminergic neurons in altered metabolic phenotypes resulting from α2-adrenergic receptor activation, we conducted experiments that assessed food intake and energy expenditure following guanabenz treatment after dopaminergic neuron inactivation using a chemogenetic technique (Figure 5A). The infection of dopaminergic neurons with the hM4D virus was confirmed by observing both green fluorescence, expressed by DAT-positive neurons, and red fluorescence produced by the hM4D virus, using fluorescence microscopy (Figure 5B). Three weeks after virus injection, mice were intraperitoneally administered clozapine N-oxide (CNO) to suppress dopaminergic neuron activity, followed by guanabenz treatment to explore the relationship between hypothalamic dopaminergic neurons and α2-adrenergic receptors in hypothalamic control of energy metabolism. The guanabenz-induced increase in food intake was significantly reversed by suppressing dopaminergic neuron activity (Figure 5C). However, no significant changes were observed in 24-h food intake within the same group of mice (Figure 5C). Furthermore, guanabenz-induced body weight gain was slightly reversed by the inactivation of dopaminergic neurons in the hypothalamic ARC 6-h period post-injection (Figure 5D). However, there were no significant differences in body weight changes within the same group of mice over a 24-h period (Figure 5D). Moreover, no changes in VO_2_ were found in mice treated with guanabenz and dopaminergic neuron inactivation compared to vehicle-treated controls (Figure 5E). However, the increase in VCO_2_, RER, and energy expenditure observed after guanabenz treatment did not occur when dopaminergic neuronal activity was suppressed (Figure 5F–H). These results support the hypothesis that hypothalamic dopaminergic neurons mediate altered metabolic phenotypes triggered by activation of α2-adrenergic receptor signaling.

## 3. Discussion

The present study highlights the significant role of dopaminergic neuron activity, modulated by α2-adrenergic receptors in the hypothalamus, in the regulation of food intake and energy expenditure. Initially, we confirmed the expression of α2-adrenergic receptors in hypothalamic dopaminergic neurons and their activation by guanabenz, an α2-adrenergic receptor agonist. Our results suggest that the activation of α2-adrenergic receptors by guanabenz may contribute to a short-term increase in food intake and body weight without significant changes over a 24-h period. This initial increase in food intake and weight gain indicates the immediate effects of α2-adrenergic signaling on hypothalamic mechanisms that control appetite. These transient effects could be attributed to several possible mechanisms, such as rapid receptor desensitization, compensatory neuroendocrine feedback loops, or circadian regulatory processes that counteract the initial pharmacological actions.

One of the key observations in this study was the significant increase in energy expenditure after guanabenz administration. This is characterized by an elevated RER, indicating increased glucose utilization. Increased RER suggests that the metabolic effects of α2-adrenergic receptor activation may involve a change in carbohydrate metabolism, which is consistent with the observed increase in food intake. The role of adrenergic signaling in energy expenditure is well documented, particularly through β-adrenergic receptors, which enhance both energy expenditure and fat metabolism [18,19]. However, increases in food intake and energy expenditure after guanabenz administration do not align with typical metabolic patterns induced by homeostatic factors that regulate energy metabolism. AgRP and POMC neurons maintain energy homeostasis by modulating food intake and energy expenditure in opposite directions. The activation of AgRP neurons increases food intake and decreases energy expenditure, while activated POMC neurons suppress food intake and increase energy expenditure [20,21,22]. Our study extends this understanding by suggesting that α2-adrenergic receptor signaling may influence energy balance through dopaminergic pathways.

Previous studies have reported that activation of dopaminergic neurons in the ventral tegmental area also increases food intake and energy expenditure [23,24], paralleling our findings induced by α2-adrenergic receptor activation. Recent studies have identified the involvement of hypothalamic dopaminergic neurons in hunger signaling, leading to increased food intake and inhibition of POMC neurons, thus contributing to the regulation of energy homeostasis [14,15]. Our findings suggest that hypothalamic dopaminergic neurons may partially mediate the guanabenz-induced increase in energy expenditure and associated metabolic changes. This conclusion is supported by a significant reduction in guanabenz-induced food intake and energy expenditure when dopaminergic neuronal activity is chemogenetically inhibited. However, reducing dopaminergic neuronal activity did not completely eliminate the enhancing effect of guanabenz on food intake, indicating the involvement of alternative pathways. Other studies have shown the presence of α2-adrenergic receptors in AgRP neurons, where norepinephrine treatment activates AgRP neurons and inhibits POMC neurons [25]. Our findings, along with these previous studies, suggest that α2-adrenergic receptors do not only regulate feeding behavior through dopaminergic neurons but instead modulate the activity of various target neurons in the hypothalamus, thus regulating feeding behavior. Interestingly, the guanabenz-induced increase in RER and energy expenditure was abolished in mice with reduced activity of hypothalamic dopaminergic neurons. These findings suggest that hypothalamic dopaminergic neurons may serve as a major neuronal population that mediates the increase in energy expenditure induced by α2-adrenergic receptor activation. Although our study primarily focuses on the role of α2-adrenergic receptors in hypothalamic dopaminergic neurons, the regulation of energy balance involves broader neural circuits beyond these specific neurons. Notably, dopaminergic populations in the ventral tegmental area (VTA), one of the major centers of dopamine production, have also been implicated in the regulation of feeding behavior and energy metabolism [26,27,28]. In this context, it is possible that dopaminergic neurons in the VTA may also be modulated by α2-adrenergic signaling. Furthermore, given that α2-adrenergic receptors are known to be expressed in AgRP neurons and other appetite-regulating neuronal populations, functional interactions between α2-adrenergic receptor signaling and these circuits may contribute to the integrated regulation of whole-body metabolic processes.

Another key aspect of our findings is their potential therapeutic implications. Given the roles of α2-adrenergic receptors and hypothalamic dopaminergic neurons in energy balance regulation, targeting these pathways may provide new strategies for managing metabolic diseases, such as obesity, and disease-induced eating disorders, including cancer cachexia. Our study highlights the need to explore novel approaches that modulate α2-adrenergic receptors for targeted interventions.

While our findings provide novel insight into the involvement of α2-adrenergic receptors and hypothalamic dopaminergic neurons in metabolic regulation, further studies will be necessary to validate and expand upon these results. Further investigations using complementary approaches, such as the application of alternative α2-adrenergic agonists, genetic disruption of α2-adrenergic receptor signaling, and experimental designs with increased sample sizes, may help clarify the underlying mechanisms and improve the generalizability of the findings. In particular, elucidating the intracellular signaling pathways by which α2-adrenergic receptors influence dopaminergic neuronal activity remains an important direction for further research.

## 4. Materials and Methods

### 4.1. Animals

Eight-week-old C57BL/6 male mice with an initial body weight of 22 ± 2 g were purchased from Dae Han Bio Link (Eumseong, Republic of Korea). Transgenic mice (DAT-Cre [Stock No. 006660], AgRP-Cre [Stock No. 012899], and Rosa26-EGFP [Stock No. 005572]) were obtained from Jackson Laboratory (Bar Harbor, ME, USA). DAT-Cre mice were crossbred with Rosa26-EGFP mice to label DAT-expressing cells with EGFP (DAT-Cre; EGFP mice). All animals were kept in a temperature- and humidity-controlled room with a 12 h-12 h light-dark cycle, with lights on from 7:00 am to 7:00 pm. All animal care and experimental procedures were performed in accordance with the protocols approved by the Institutional Animal Care and Use Committee (IACUC) of Incheon National University (permission number: INU-ANIM-2021-01; approval date: 4 May 2021).

### 4.2. Administration of Norepinephrine or Guanabenz and Measurement of Food Intake and Body Weight

For intracerebroventricular (i.c.v.) implantation of the cannula, mice were anesthetized by an intraperitoneal (i.p.) injection of tribromoethanol (250 mg/kg, Sigma-Aldrich, St. Louis, MO, USA) and placed in a stereotaxic apparatus (Stoelting, Wood Dale, IL, USA). A 26-gauge cannula was surgically implanted in the right lateral ventricle, targeting coordinates of 1.0 mm lateral, 0.3 mm posterior, and 2.4 mm ventral relative to the bregma, as per the Stereotaxic Mouse Brain Atlas (Paxinos G and Franklin KBJ, 2001, Academic Press, San Diego, CA, USA). The cannula was anchored to the skull using dental cement. Following surgery, the mice were kept warm until they fully recovered from anesthesia and were then housed individually in separate cages. After 7 days of recovery, the mice were injected with saline, norepinephrine (300 nmol in 2 μL at 0.5 μL/min rate, Sigma-Aldrich), or guanabenz (100 ng in 2 μL at 0.5 μL/min rate, G110, Sigma-Aldrich). Body weight and food intake were measured 1, 2, 3, 4, 5, 6, and 24 h after injection.

### 4.3. Measurement of O_2_ Consumption, CO_2_ Production, and Energy Expenditure

Oxygen consumption (VO_2_), carbon dioxide production (VCO_2_), and energy expenditure in mice were assessed using an indirect calorimetry system (Promethion, Sable Systems, Las Vegas, NV, USA). Food and water were freely accessible to the animals. Before data collection, the mice were acclimated to the metabolic cages for 48 h under a 12-h light/dark cycle. VO_2_ and VCO_2_ were recorded for each mouse at 10-min intervals. The respiratory exchange ratio (RER) was determined by calculating the ratio of VCO_2_ to VO_2_. Data acquisition and system control were managed with MetaScreen software (version 2.3.12), and the raw data were analyzed with Expe-Data software (version 1.9.14, Sable Systems).

### 4.4. Immunohistochemistry

DAT-Cre; EGFP mice were deeply anesthetized with tribromoethanol (i.p., 250 mg/kg, Sigma-Aldrich) 90 min after injection of norepinephrine or guanabenz. Transcardiac perfusion was performed with ice-cold 0.9% saline and 3% paraformaldehyde in 0.1 M phosphate buffer (PB, pH 7.4). The brains were extracted and post-fixed in 3% paraformaldehyde at 4 °C overnight. Coronal sections (thickness, 50 μm) were prepared using a vibratome (5100 mz, Campden Instruments Ltd., Leicestershire, UK). The coronal sections were incubated with 0.01 mol/L citrate buffer for 10 min at 80 °C and then rinsed in PB at room temperature (RT). The sections were pre-incubated with 0.2% Triton X-100 (Sigma-Aldrich) in PB for 30 min at RT. After several washes with PB, the sections were incubated overnight at 4 °C with rabbit c-Fos antibody (1:1000, Cell signaling technology, Danvers, MA, USA). The next day, the sections were washed with PB for 30 min and incubated at RT with goat anti-rabbit Alexa Fluor 594 secondary antibody (1:500, A11012, Invitrogen, Carlsbad, CA, USA). The sections were then mounted on glass slides and the coverslips were placed on the slides with a drop of mounting medium (Dako, North America Inc., Carpinteria, CA, USA). Coverslips were sealed with nail polish to prevent drying and movement of samples under the microscope.

### 4.5. Image Capture and Analysis

Fluorescence microscopy (Axioplan2 Imaging; Carl Zeiss Microimaging Inc., Oberkochen, Germany) was used to capture images of c-Fos- and DAT-positive cells. For immunohistochemistry (IHC) analysis, brain sections were aligned with a mouse brain atlas for anatomical accuracy. The dopaminergic neurons and c-Fos-positive cells were quantified using ImageJ software version 1.47v (National Institutes of Health, Bethesda, MD, USA; https://imagej.nih.gov/ij/; accessed on 20 March 2021) by an independent observer to ensure unbiased counts.

### 4.6. Electrophysiology

Whole-cell patch-clamp recordings were performed on DAT-EGFP neurons in hypothalamic slices. Brains were quickly removed and immersed in ice-cold, carbogen-saturated (95% O_2_, 5% CO_2_) sucrose solution containing 75 mM sucrose, 2.5 mM KCl, 7 mM MgCl_2_, 0.5 mM CaCl_2_, 1.3 mM NaH_2_PO_4_, 25 mM NaHCO_3_, and 25 mM glucose at pH 7.3. Coronal slices of the hypothalamus (300 μm thick) were prepared in a sucrose solution using a vibratome (Leica VT1200, Leica Biosystems, Wetzlar, Germany). The slices were then transferred to a chamber filled with oxygenated artificial cerebrospinal fluid (ACSF) containing 125 mM NaCl, 2.5 mM KCl, 1 mM MgCl_2_, 2 mM CaCl_2_, 1.3 mM NaH_2_PO_4_, 25 mM NaHCO_3_, and 25 mM glucose at pH 7.3, and incubated at room temperature for at least 1 h before recording. For recordings, the slices were placed in a recording chamber perfused with ACSF (continuously bubbled with 95% O_2_, 5% CO_2_) at a flow rate of ~3 mL/min, maintained at 30–32 °C. Recording pipettes, with a tip resistance of 5–9 MΩ, were filled with an internal solution containing 135 mM K-gluconate, 5 mM KCl, 1 mM MgCl_2_, 0.02 mM CaCl_2_, 0.2 mM EGTA, 10 mM HEPES, 4 mM Na_2_-ATP, and 0.3 mM Na-GTP (pH adjusted to 7.3 with NaOH, 290–293 mOsm). Borosilicate glass pipettes were prepared using a PC-10 vertical puller (Narishige, Tokyo, Japan). DAT-positive neurons in the arcuate nucleus were visualized using a BX51WI fluorescence microscope (Olympus, Tokyo, Japan) with a Zyla 5.5 CCD camera (Andor, Abingdon, UK), and differential interference contrast was applied to form a seal on the cells. After achieving a gigaohm seal, gentle suction was applied to attain a whole-cell configuration. Guanabenz (30 μM) was added to the ACSF and perfused for 3–5 min during recordings. All recordings were conducted with a Multiclamp 700B amplifier (Molecular Devices, San Jose, CA, USA). Data were collected via pCLAMP software (version 2.2.2.2, Molecular Devices), and the mean firing rate and membrane potential were analyzed with Clampfit software (version 10.7.0.3, Axon Instruments Inc., Union City, CA, USA).

### 4.7. Robo-Tag Assays

To analyze DAT-or AgRP neuron–specific gene expression, we used the Ribo-Tag translational profiling system in Rpl22^HA^ mice (Stock No. 011029, Jackson Laboratory) [29,30]. Because Rpl22HA mice have loxP-flanked WT exon 4 followed by an identical exon 4 tagged with hemagglutinin A (HA), floxed WT exon 4 was replaced with HA-tagged exon 4 in cells expressing Cre recombinase. To generate ribo-tagged mice (DAT-Cre; Rpl22^HA^ or AgRP-Cre; Rpl22^HA^), we crossbred Rpl22^HA^ mice with DAT-Cre or AgRP-Cre. DAT-Cre; Rpl22^HA^ or AgRP-Cre; Rpl22^HA^ mice specifically expressed Rpl22-tagged with the HA protein in the ribosomes of DAT and POMC neurons, respectively. RNA was isolated using the Ribo-Tag system as previously described [31]. The hypothalamus was collected and homogenized, with RNA extracted from 10% of the cleared lysate to serve as the input control. The remaining lysate was incubated with mouse anti-HA antibodies for 4 h at 4 °C, followed by protein G agarose bead (LGP-1018B, Lugen SCI, Gyeonggi-Do, South Korea) addition and overnight incubation at 4 °C. After three washes in a high-salt solution, bound ribosomes and RNA were released from the beads by 30 s of vortexing. Total RNA was isolated using the QIAGEN RNeasy Micro Kit (74034; Qiagen, Hilden, Germany) following the manufacturer’s protocol, and its concentration was measured with a NanoDrop Lite (Thermo Scientific, Waltham, MA, USA). For assessing ribosome-associated mRNA levels, cDNA was synthesized using a high-capacity cDNA reverse transcription kit (Applied Biosystems, Foster City, CA, USA), followed by quantitative real-time PCR (qRT-PCR) analysis.

### 4.8. Quantitative Real-Time PCR

Ribosome-associated mRNA expression levels were quantified using a Bio-Rad CFX 96 Real-Time Detection System (Bio-Rad Laboratories, Hercules, CA, USA) with a SYBR Green Real-time PCR Master Mix Kit (TaKaRa Bio Inc., Foster, CA, USA). Data analysis was conducted with CFX Manager software (version 3.1, Bio-Rad Laboratories), and expression levels were normalized to the housekeeping genes *β-actin* and *L19* for consistency. The primers used were as follows: *AgRP*, F-CTCCACTGAAGGGCATCAGAAG and R-ACTCAGCACCTCCGCCAAAG; *DAT*, F-TTGCAGCTGGCACATCTATC and R-ATGCTGACCACGACCACATA; *α2A*, F-TTCTTTTTCACCTACACGCTCA and R-TGTAGATAACAGGGTTCAGCGA; *α2B*, F-ACCTTCCCTTGCTGACTGTACT and R-TGGGAGGGAGGTATTCTAATCA; *α2C*, F-GGCTGTGAACTTAGGGCTTTAG and R-ATAGGAAGTCAGCCCTTGCTC; *L19*, F-GGTGACCTGGATGAGAAGGA and R-TTCAGCTTGTGGATGTGCTC; *β-actin,* F-GATCTGGCACCACACCTTCT and R-GGGGTGTTGAAGGTCTCAAA.

### 4.9. Virus Injection and Clozapine N-Oxide (CNO) Treatment

DAT-Cre; EGFP mice were anesthetized with tribromoethanol (250 mg/kg; Sigma-Aldrich) and placed on a stereotactic apparatus (Stoelting). pAAV-hSyn-DIO-mCherry (titer: 4.7 × 10^12^ GC/mL, Addgene: 50459-AAV2, MA, USA) and pAAV-hSyn-DIO-hM4D(Gi)-mCherry (titer: 1.4 × 10^13^ GC/mL, Addgene: 44362-AAV2) were used as Cre-dependent designer receptors exclusively activated by designer drug (DREADD) expression. Virus was injected into the hypothalamic arcuate nucleus (ARC) (AP, 1.7 mm; ML, ±0.28 mm; DV, 6.02 mm from bregma; 1 uL/10 min). The animals were allowed to recover from surgery for 3 weeks. For behavioral tests, 3 mg/kg of CNO in saline was injected through a small gauge (32) syringe once 30 min before the test.

### 4.10. Statistical Analysis

Statistical analyses were performed using GraphPad Prism 9 software (GraphPad Software, San Diego, CA, USA). All data are expressed as mean ± SEM. Statistical significance was determined using an unpaired two-tailed Student’s *t*-test for normally distributed data. One-way ANOVAs were used for comparisons involving more than two groups. For multiple comparisons, a post hoc analysis using Sidak’s test was performed to determine differences between groups. Detailed statistical methods for each experiment are presented in the figure legends.

## 5. Conclusions

This study elucidates the critical interaction between α2-adrenergic receptors and hypothalamic dopaminergic neurons in the regulation of feeding behavior and energy expenditure. Although the actions of α2-adrenergic receptors are significantly mediated by hypothalamic dopaminergic neurons, the involvement of other neuronal pathways and compensatory mechanisms in the regulation of energy homeostasis modulated by α2-adrenergic signaling is also implied. Future research should focus on the chronic effects of α2-adrenergic receptor activation, possible intervention strategies, and the crosstalk between adrenergic signaling and other metabolic pathways in the hypothalamus. These efforts will improve our understanding of whole-body metabolic regulation and potentially lead to novel therapeutic approaches to metabolic disorders.

## Data Availability

The original contributions presented in this study are included in the article. Further inquiries can be directed to the corresponding authors.
